# Spontaneous Regression of Locally Advanced Breast Cancer Following Cardiopulmonary Arrest: A Case Report

**DOI:** 10.7759/cureus.78111

**Published:** 2025-01-28

**Authors:** Asahi Kannari, Masayuki Kikuchi, Hirokazu Matsushima, Rika Miyabe, Koji Atsuta

**Affiliations:** 1 Surgery, Japanese Red Cross Shizuoka Hospital, Shizuoka, JPN; 2 Surgery, Tosen Clinic, Shizuoka, JPN

**Keywords:** bleeding tumor, breast cancer, cancer management, cardiac pulmonary arrest (cpa), locally advanced breast-cancer, spontaneous regression, spontaneous regression of cancer

## Abstract

Spontaneous regression in breast cancer is rare but can dramatically improve patient prognosis. Although the underlying mechanism is unknown, it may be due to a biological response to external invasion. An 81-year-old woman presented to our emergency department with a 600x100mm large breast mass. Five days after the emergency room visit, she lost consciousness bleeding from the breast mass. She experienced cardiopulmonary arrest (CPA), and after 10 minutes of cardiopulmonary resuscitation, the patient underwent a return of spontaneous circulation (ROSC). She was diagnosed with hemorrhagic and cardiogenic shock, and the breast mass gradually collapsed on the 17th day. Twelve months after CPA, the patient underwent left mastectomy and axillary lymph node dissection (II) for left breast cancer. Postoperatively, the patient continued to receive aromatase inhibitors and radiation therapy and she did not experience any recurrence two years after surgery. Spontaneous regression of breast cancer following CPA has not been previously reported, and, to the best of our knowledge, this case report is the first. We hypothesized that the tumor might have had relative ischemia and internal necrosis due to the blockage of the nutrient artery.

## Introduction

Spontaneous regression is defined as “the phenomenon of partial or complete disappearance of a tumor without any known effective treatment" [1]. It is rare, ranging from 60,000 to 100,000 for all malignancies combined, and has generally been reported in renal cell carcinoma, malignant melanoma, neuroblastoma, and hepatocellular carcinoma [2]. Of those, only 30 cases of breast cancer have been reported to date [3]. The spontaneous regression of breast cancer, as with other tumors, often involves events occurring immediately before regression [1,3]. In breast cancer cases, regression triggered by surgical invasion has been reported. However, the pathology underlying breast cancer regression remains unclear. Here, we report a rare case of rapid spontaneous regression of locally advanced breast cancer after cardiopulmonary arrest (CPA).

## Case presentation

**Figure 1:**
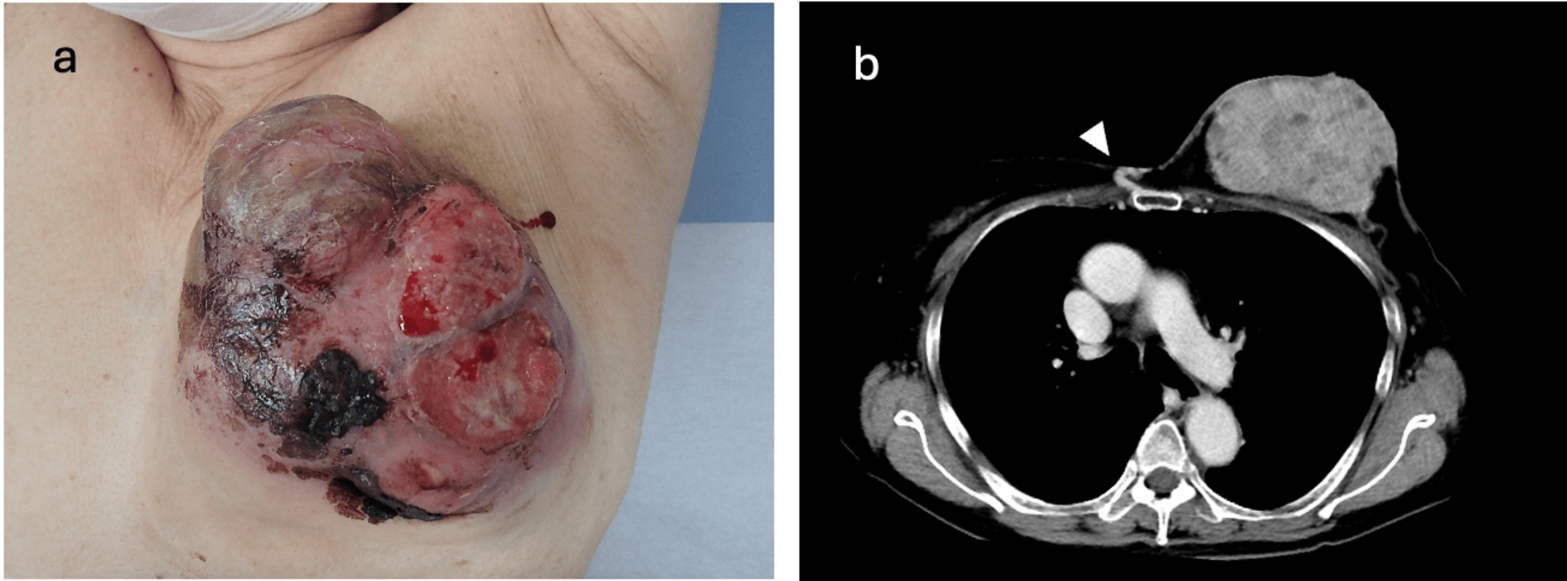
(a) Patient photograph taken before the cardiopulmonary arrest (CPA). (b) Contrast-enhanced CT scan showing a 600 × 100 mm mass. The arrowhead indicates the left internal mammary artery.

An 81-year-old woman presented to our emergency department with lightheadedness and bleeding from a left breast mass. Her medical history included cholecystitis, uterine fibroids, and right lower extremity paralysis. When she visited our hospital, her consciousness was clear, blood pressure was 151/98 mmHg, pulse was 86/min, and oxygen saturation was 100% (room air). The mass in the left breast was mobile. It showed persistent venous bleeding, which was stopped by compression (Figure 1a). Blood tests revealed a hemoglobin (Hb) level of 12.0 g/dL, a carcinoembryonic antigen (CEA) level of 7.21 ng/mL, and a cancer antigen 15-3 (CA 15-3) level of 73 U/mL (Table 1). Contrast-enhanced computed tomography (CT) showed a 600 x 100 mm mass with a well-defined border and a contrast effect in the left breast (Figure 1b). A needle biopsy revealed invasive ductal carcinoma of the breast, characterized by estrogen receptor (ER) positivity at 60%, progesterone receptor (PR) positivity at 40%, human epidermal growth factor receptor 2 (HER2) negativity at 0%, and a Ki-67 index of 8%.

**Table 1:** Blood tests before cardiopulmonary arrest (CPA) show elevated tumor markers, including carcinoembryonic antigen (CEA) and cancer antigen 15-3 (CA 15-3), while hemoglobin levels remain within the normal range.

	Result	Reference range
Hemoglobin	12.0 g/dL	11.5-15.0 g/dL
Carcinoembryonic antigen (CEA)	7.21 ng/mL	0.0-1.5 ng/mL
Cancer antigen 15-3 (CA 15-3)	73 U/mL	0.00-5.00 U/mL

**Figure 2:**
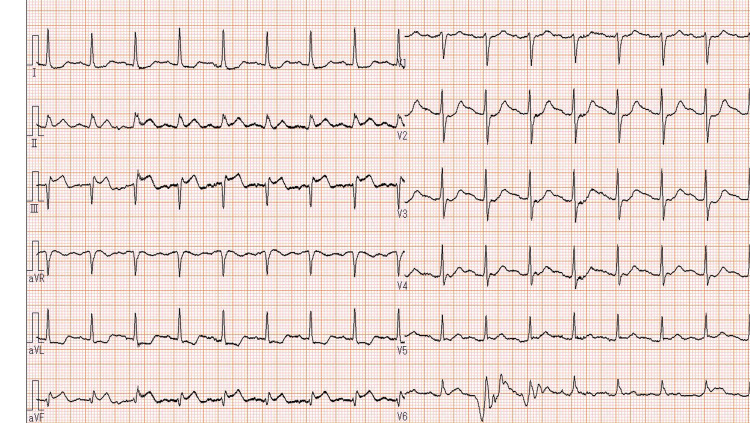
Post-resuscitation ECG showing ST-segment elevation in leads II, III, and aVF (V6 lead: electrocardiogram artifact).

However, five days after the emergency room visit, the patient was rushed to the emergency room because of loss of consciousness due to massive bleeding from the breast tumor. She experienced CPA immediately before arrival at the emergency room, and cardiopulmonary resuscitation (CPR) was immediately performed. After the patient arrived at the hospital, an electrocardiogram (ECG) revealed pulseless electrical activity, and after 10 minutes of CPR, the patient underwent a return of spontaneous circulation (ROSC). Blood results at the time of CPA showed Hb of 8.6 g/dL and troponin T of 0.068 ng/mL (Table 2). Post-resuscitation ECG showed ST-segment elevation in leads II, III, and aVF (Figure 2), and the patient was judged to have a right ventricular infarction. She was diagnosed with hemorrhagic and cardiogenic shock.

**Table 2:** Blood test after cardiopulmonary arrest (CPA) shows anemia and elevated myocardial deviation enzymes.

	Result	Reference range
Hemoglobin	8.6 g/dL	11.5-15.0 g/dL
Troponin T	0.068 ng/mL	0.00-0.014 ng/mL

After ROSC, the patient was managed with a ventilator and intensive care with norepinephrine and blood transfusions. The cardiologist decided to perform a coronary angiography (CAG) after the patient's general condition had stabilized. Upper gastrointestinal endoscopy was performed one day after admission, and no obvious gastrointestinal bleeding was identified. The patient was weaned from the ventilator five days after admission, and the breast mass suddenly collapsed on the 17th day.

**Figure 3:**
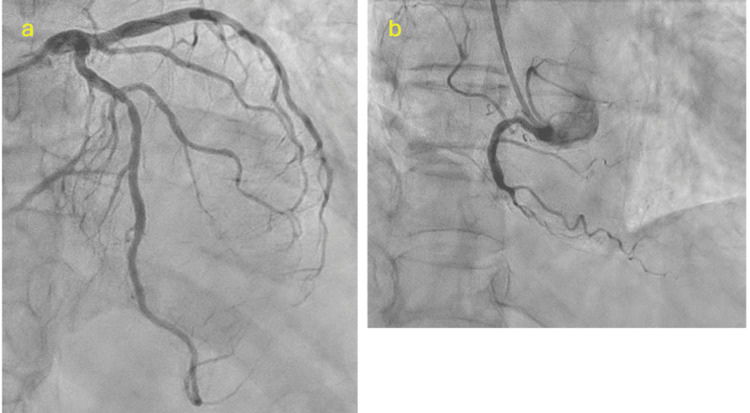
(a) Coronary angiogram in posterior-anterior (PA)-cranial 40° projection showing 50% stenosis in the mid-left anterior descending (LAD) artery. (b) Coronary angiogram in PA-cranial 20° projection showing chronic total occlusion of the right coronary artery (RCA).

**Figure 4:**
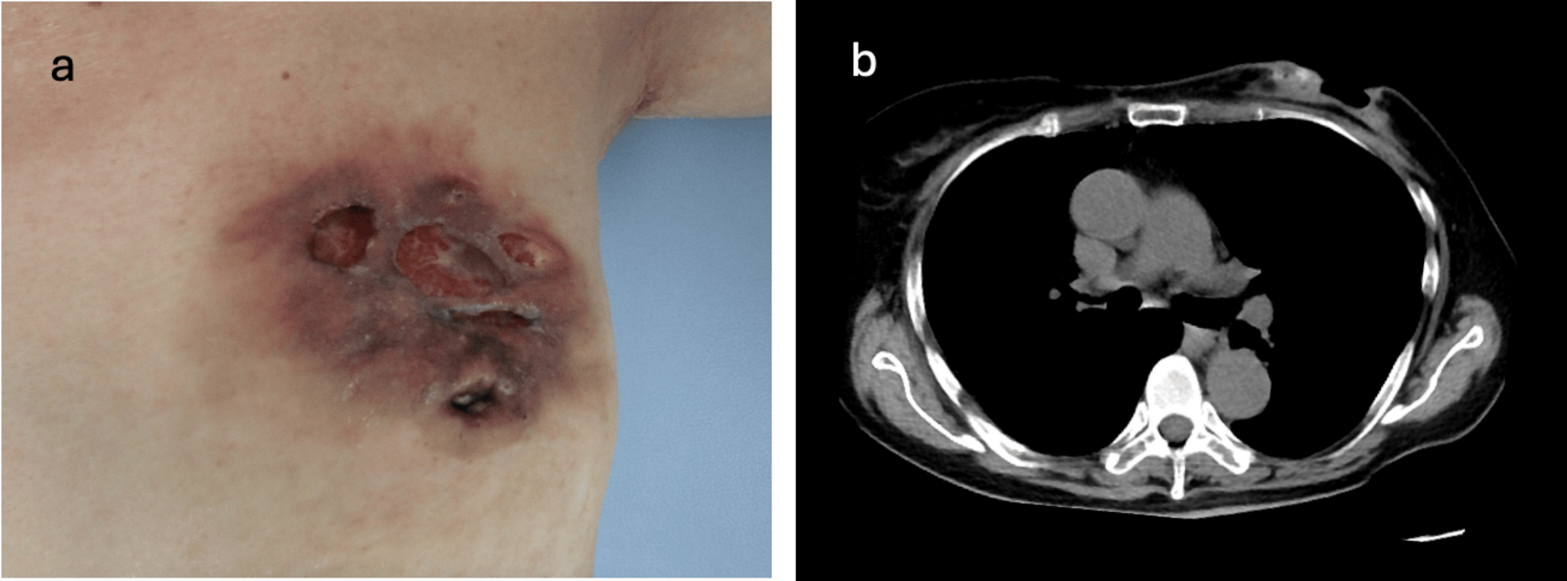
(a) Patient photograph taken 37 days after cardiopulmonary arrest (CPA). (b) CT images showing tumor regression to 40 mm.

On the 21st day, CAG was performed, which revealed chronic total occlusion of the right coronary artery (Figure 3). Because it was a chronic total occlusion and the patient's hemodynamics were stable, urgent stenting was deemed unnecessary. Fifteen milligrams of nicorandil, 100 mg of diltiazem, and 10 mg of pravastatin/day were started on the 22nd day. Blood pressure recovered, and the patient was able to walk on her own. She was discharged from the hospital on the 30th day. CT performed on day 37 revealed that the tumor had regressed to 40 mm (Figures 4a, 4b). After explaining the treatment plan to the patient, we decided to start hormonal therapy using letrozole (an aromatase inhibitor) first, followed by surgery.

Two months after discharge, percutaneous coronary stenting was performed for chronic complete occlusion of the right coronary artery (75 × 23 mm and 3.0 × 28 mm; Xience Skypoint 2, Plymouth, MN, USA). Blood results five months after the CPA showed a CEA level of 3.98 ng/mL and a cancer antigen 15-3 (CA 15-3) level of 13 U/mL (Table 3). Twelve months after CPA, the patient underwent left mastectomy and axillary lymph node dissection (II) for left breast cancer. The pathological diagnosis was invasive ductal carcinoma of the breast, with ER positivity at 100%, PR positivity at 5%, and HER2 scored as 2+. The pathological TNM classification was pT4NxM0, corresponding to Stage IIIB. The patient had a good postoperative course and was discharged on postoperative day 6. Postoperatively, the patient continued to receive letrozole and radiation therapy (50 Gy/postmastectomy radiation therapy for one month, from 18 months to one year) and did not experience any recurrence for two years after the surgery.

**Table 3:** Blood tests five months after cardiopulmonary arrest (CPA) show a decrease in carcinoembryonic antigen (CEA) and cancer antigen 15-3 (CA 15-3) levels.

	Result	Reference range
Carcinoembryonic antigen (CEA)	3.98 ng/mL	0.0-1.5 ng/mL
Cancer antigen 15-3 (CA 15-3)	13 U/mL	0.00-5.00 U/mL

## Discussion

In this case, the cause of CPA was thought to be related to multiple pathological conditions. First, there was a large amount of bleeding from the breast mass, and the amount of circulating plasma in the blood vessels markedly decreased. A key finding of this case is that the tumor shrank rapidly two weeks after CPA, despite no treatment for breast cancer administered throughout the pre- and post-resuscitation periods. This observation falls within the definition of spontaneous regression, which is “the phenomenon of partial or complete disappearance of a tumor without any known effective treatment" [1].

Spontaneous regression is rare, ranging from 60,000 to 100,000 for all malignancies combined, and has generally been reported in renal cell carcinoma, malignant melanoma, neuroblastoma, and hepatocellular carcinoma [2]. However, only 30 cases of breast cancer have been reported [3]. The pathology underlying breast cancer regression remains unclear. The spontaneous regression of breast cancer, as with other tumors, often involves events occurring immediately before regression [1,3]. In breast cancer cases, regression triggered by surgical invasion, especially needle biopsy, has been reported [3-8]. For example, De Faria Castro Fleury et al. reported a case of regression at 35 days after needle biopsy [5]. As to the reason for the regression after biopsy, Qureshi et al. postulated that the needle biopsy might disrupt the microenvironment of the remaining tumor and activate the immune response, resulting in immunological cell death [7]. In other cases, regression occurred after metformin administration [9] and herbal medicine [10]. However, rapid spontaneous regression of breast cancer following CPA has not been previously reported, and this case report is the first. Since needle biopsy was performed before CPA, we cannot exclude the possibility that the biopsy triggered the regression in this case. However, while a typical biopsy regression is interspersed within approximately 40 days, the present case shrank rapidly within two weeks after the hemorrhage. This finding led us to consider the possibility that factors other than the biopsy were responsible for the regression observed in this case.

In this retrospective comparison of images taken during the course of this case, the perforating branch of the internal thoracic artery, which nourishes the tumor, was not identified on CT before CPA but on preoperative MRI. In other words, we hypothesized that, as the background of regression, the nutrient vessels from the internal thoracic artery to the tumor may have been blocked by a mechanism following the hemorrhage. Although the direct cause of blockage of the nutrient artery is unclear, invasion during CPA or administration of catecholamines may have caused thrombosis.

In recent years, there have been case reports on transcatheter arterial embolization (TAE) for bleeding control in patients with locally advanced breast cancer [11-16]. For example, Aksoy et al. successfully saved the life of a patient with breast cancer in shock due to tumor bleeding by embolizing the internal thoracic feeder artery [11]. In several cases, the tumor shrank after TAE. Tokunaga et al. reported a case of tumor necrosis one week after embolization of nutrient vessels branching from the internal thoracic and thoracoacromial arteries in a patient with breast cancer that had recurred in the chest wall [12]. Hosono et al. also reported a case of tumor shrinkage on the day after TAE of the internal and external thoracic arteries in a patient with locally advanced breast cancer poorly controlled by chemotherapy [13].

A common feature of these cases is that the tumors regressed within a short period, from one day to one month after treatment [11-16]. This is similar to our case, and a common pathology can be expected. In other words, the tumor might have had relative ischemia and internal necrosis due to the blockage of the nutrient artery from the internal thoracic artery for some reason during CPA in this case as well. Bleeding from breast cancer is a serious complication; however, proper bleeding control is expected to result in tumor shrinkage and improve patient prognosis.

## Conclusions

To the best of our knowledge, this is the first to report spontaneous regression of breast cancer after CPA. We speculate that the tumor might have had relative ischemia and internal necrosis due to the blockage of the nutrient artery. We found that the rapid regression in this case is similar to breast tumor shrinkage after TAE, and a common pathology can be expected.
